# Opportunistic infections changed before and after SARS-CoV-2 infection in inflammatory bowel disease patients: a retrospective single-center study in China

**DOI:** 10.3389/fmed.2024.1461801

**Published:** 2024-09-24

**Authors:** Zhenzhen Fan, He Zhou, Jiaqi Zhang, Xiaoning Liu, Tong Wu, Yanting Shi, Junchao Lin, Jie Liang

**Affiliations:** State Key Laboratory of Holistic Integrative Management of Gastrointestinal Cancers and National Clinical Research Center for Digestive Diseases, Xijing Hospital of Digestive Diseases, Fourth Military Medical University, Xi’an, China

**Keywords:** SARS-CoV-2, opportunistic infection, nomogram, inflammatory bowel disease, Crohn’s disease, ulcerative colitis

## Abstract

**Objective:**

SARS-CoV-2 transmission has become a serious worldwide public health concern. However, there is currently insufficient data to determine whether SARS-CoV-2 infection would affect opportunistic infections in inflammatory bowel disease (IBD) patients.

**Methods:**

A retrospective study included 451 IBD patients (294 UC and 157 CD). The IBD patients were divided into two groups: before SARS-CoV-2 infection and after SARS-CoV-2 infection, and outcomes were measured for these groups. The primary outcome was the presence and distribution of opportunistic infections. The secondary outcomes included factors associated with opportunistic infections, based on which a nomogram prediction model was developed and validated.

**Results:**

After SARS-CoV-2 infection, the proportion of IBD patients with opportunistic infections by *Clostridium difficile* (21.31% vs. 14.01%, *p* = 0.044) and Epstein–Barr virus (13.93% vs. 4.35%, *p* = 0.001) was significantly higher compared to that before. Conversely, the proportion of patients with hepatitis B virus (3.69% vs. 10.14%, *p* = 0.006) and herpes simplex virus type I (1.23% vs. 4.35%, *p* = 0.04) infections was significantly lower after the infection. Additionally, pre-SARS-CoV-2 infection factors associated with opportunistic infections in IBD include duration of illness, red blood cell count, the presence of comorbid chronic illnesses, and alcohol consumption, while post-SARS-CoV-2 infection, the primary risk factors involve corticosteroid use, red blood cell count, protein level, and high-sensitivity C-reactive protein.

**Conclusion:**

After the SARS-CoV-2 infection, there has been a shift in the occurrence of opportunistic infections among IBD patients. It might be attributed to the use of corticosteroids and also the strengthening of containment measures, heightened public health awareness, and widespread vaccination.

## 1 Introduction

Inflammatory Bowel Disease (IBD) is a chronic, relapsing autoimmune disorder with incompletely understood etiology and pathogenesis, primarily comprising Ulcerative Colitis (UC) and Crohn’s Disease (CD) (1). With no definitive cure available, treatment often relies on immunosuppressive medications, increasing the risk of opportunistic infections in patients (2). These infections are typically caused by microorganisms that have limited or no pathogenicity in healthy individuals but can cause disease when the immune function is compromised (3). Besides medication, the risk of infection is influenced by several factors, such as malnutrition, old age, genetic immunodeficiencies, and other chronic conditions. While the direct correlation with immunosuppressive drugs is established, the impact of other factors is supported mainly by indirect evidence (4). IBD patients frequently contract the following opportunistic infections (1): viral infections, such as cytomegalovirus (CMV), Epstein–Barr virus (EBV), herpes simplex virus type I (HSV-I), herpes simplex virus type II (HSV-II), hepatitis B virus (HBV), hepatitis C virus (HCV), and rubella virus (RV) (2); bacterial infections, like TB and *Clostridium difficile* infection (CDI) (3); fungal infections, like Candida (4); parasitic infections, like Toxoplasma (TOX) (2, 5, 6).

Against the backdrop of the SARS-CoV-2 pandemic (7), this virus not only impacts the respiratory system but also affects multiple organs including the heart, liver, gastrointestinal system (8–10). Research by Zhong et al. has also shown that SARS-CoV-2 infection can cause gastrointestinal symptoms like diarrhea, nausea, vomiting, and loss of appetite (11). This is particularly significant for IBD patients, as clinical data suggest that while the disease activity and endoscopic evaluations do not show significant changes pre and post-SARS-CoV-2 infection (12), the persistent presence of viral antigens in their gastrointestinal tract and significant microbiome shifts compared to healthy controls may increase their risk of opportunistic infections (13). These findings underscore the importance of comprehensive management and close monitoring of IBD patients after SARS-CoV-2 infection.

On the other hand, IBD patients with a higher risk of opportunistic infections have been a key focus in studies on the safety of SARS-CoV-2 vaccines. Current research indicates that vaccination has not been associated with severe adverse events (14). As public health awareness increases and vaccine coverage expands, the impact of opportunistic infections on IBD patients and their prognosis may have changed. However, clinical evidence remains limited. While a few studies have addressed this issue (15), specific research on opportunistic infections is still lacking. Therefore, our study aims to explore the distribution and influencing factors of opportunistic infections among IBD patients before and after SARS-CoV-2 infection, intending to develop a predictive model for early identification and intervention of potential infection risks. We conducted a retrospective study at China’s largest IBD center, assessing the incidence and associated factors of opportunistic infections before and after infection, and based on this, developed a stable predictive model.

## 2 Materials and methods

### 2.1 Study design and participants

In this retrospective study, we collected and analyzed demographic and clinical data of patients with IBD who visited the Department of Gastroenterology at the First Affiliated Hospital of the Air Force Medical University in China from January 2020 to December 2023. Since the outbreak of SARS-CoV-2 in January 2020 and the implementation of comprehensive and strict control measures in China, all hospitalized patients were required to provide proof of negative SARS-CoV-2 nucleic acid testing. By December 2022, with the comprehensive lifting of the epidemic situation in China, the relevant policies were lifted. Therefore, this study used December 2022 as the dividing point between before and after SARS-CoV-2 infection for the included patients, dividing them into two groups: pre-infection (January 2020 to December 2022, 207 cases) and post-infection (December 2022 to December 2023, 244 cases). Subsequently, subgroup analyses for UC and CD were performed for these two groups of patients.

The First Affiliated Hospital Ethics Committee of Air Force Medical University, China gave the study their blessing (KY20232246-C-1). The study was retrospective in nature; therefore, patient consent documents were not required. This investigation was carried out in compliance with the guidelines provided in the World Medical Association’s Declaration of Helsinki.

### 2.2 Inclusion and exclusion criteria

**Figure 1:**
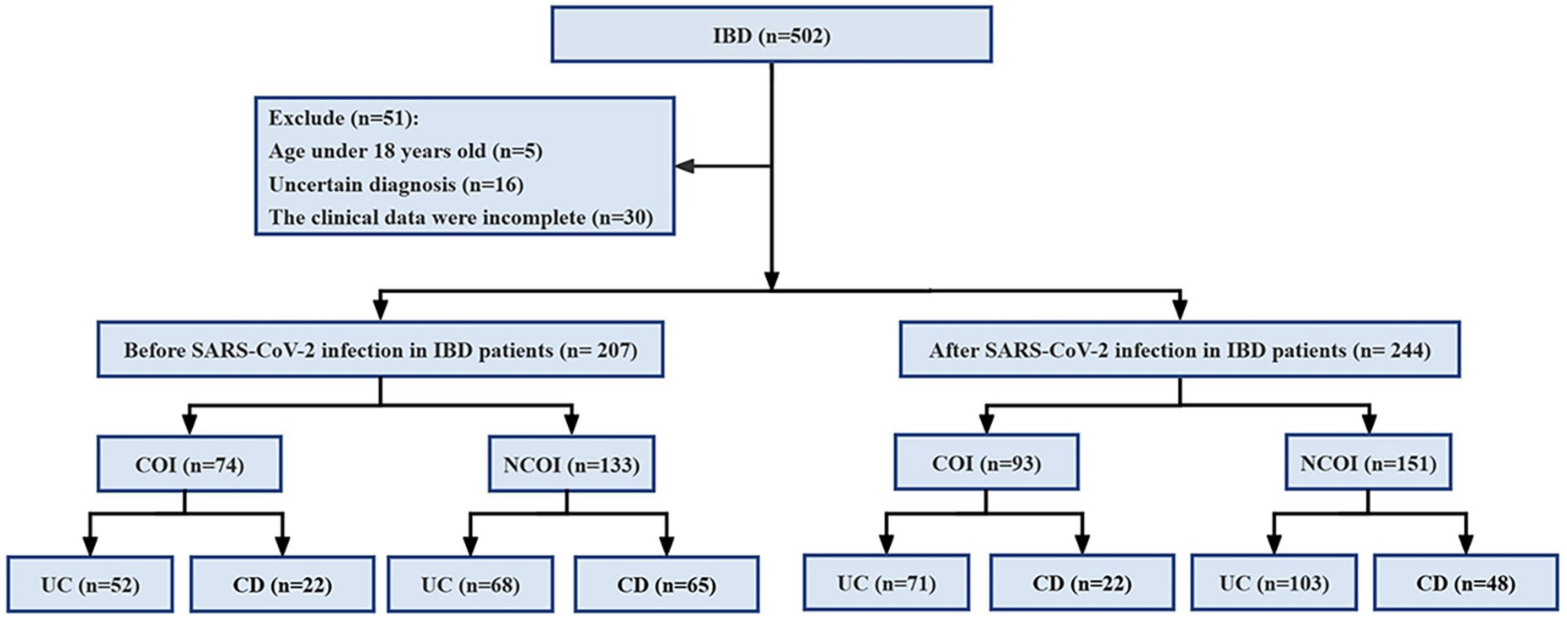
Research process diagram. IBD, inflammatory bowel disease; UC, ulcerative colitis; CD, Crohn’s disease; COI, combined opportunistic infections; NCOI, not combined opportunistic infections.

Patients eligible for the study must be aged ≥18 years, of any gender, and must have a confirmed diagnosis of IBD according to the Chinese consensus on diagnosis and treatment of inflammatory bowel disease (16), which includes clinical presentation, laboratory test results, colonoscopy and biopsy pathology, as well as imaging studies. However, patients with incomplete clinical data, those aged <18 years, unclear diagnosis of IBD, or non-primary diagnosis of IBD were excluded from the analysis. A total of 502 patients were included in this study, with 51 excluded because they were aged <18 years (*n* = 5), unable to provide a clear diagnosis (*n* = 16), or had incomplete clinical data (*n* = 30; Figure 1).

### 2.3 Collection of clinical data

This study systematically gathered baseline clinical data from IBD patients, encompassing demographic parameters (disease type, gender, age, height, weight, BMI, smoking history, and alcohol consumption), underlying chronic conditions (such as hypertension, diabetes, chronic liver disease, chronic heart disease, chronic kidney disease, chronic lung disease, and chronic gastritis), extraintestinal manifestations (like joint pain, iritis or uveitis, erythema nodosum, pyoderma gangrenosum, oral ulcers, hepatobiliary disease, and thromboembolic disease), complications (psoriasis, toxic megacolon, intestinal perforation, lower gastrointestinal bleeding, dysplasia, malignancy, fistula, perianal lesions, and ankylosing spondylitis), disease progression, medication history for IBD (including 5-Aminosalicylic Acid, corticosteroids, immunosuppressants, and biologics), Montreal classification, IBD staging (mild, moderate, severe, and remission), opportunistic infections (CMV, EBV, HBV, HCV, HSV-I, HSV-II, RV, TOX, TB, CDI, and fungi), and laboratory test indicators, in addition to SARS-CoV-2 testing.

### 2.4 Definition of combined opportunistic infections

The opportunistic infection guidelines for Crohn’s disease and ulcerative colitis in Europe (17) and the evidence-based agreement on opportunistic infections in Chinese inflammatory bowel disease (18) determine which pathogens are chosen for examination in this study. Here’s how one defines combined opportunistic infections: (1) HBV infection: peripheral blood HBsAg positive or HBV-DNA >100 IU/ml; (2) HCV infection: peripheral blood anti-HCV IgM/IgG positive or elevated HCV-RNA copy number; (3) HSV infection: clinical symptoms (clustered vesicles and herpes-like skin lesions, etc.) combined with HSV-IgM positive or positive nucleic acid test in lesion tissue or secretion to confirm diagnosis; (4) CMV infection: clinical symptoms (watery diarrhea or bloody diarrhea, abdominal pain, etc.) combined with elevated CMV-DNA copy number or positive pp65 antigen or positive CMV IgM or positive inclusion bodies to confirm diagnosis; (5) EBV infection: clinical symptoms (fever, pharyngitis, lymphadenopathy, etc.) combined with laboratory tests (positive EBV IgM or positive EBV nuclear antigen or elevated peripheral blood EBV-DNA copy number) to confirm diagnosis; (6) TB infection: diagnosis based on previous history of tuberculosis, contact history, chest X-ray, tuberculin skin test (PPD skin test), and interferon release assay (IGRAs); (7) CDI: diagnosed based on clinical symptoms such as watery diarrhea, abdominal pain, and positive detection of CDI toxin B in feces or amplification of toxin B gene through nucleic acid amplification test, or positive fecal culture; (8) Fungal infection: diagnosed based on clinical symptoms of fungal infection and positive laboratory tests (fungal culture or pathology). Some patients may also show typical fungal infection imaging manifestations, and positive serum G test/GM test can assist in diagnosis; (9) SARS-CoV-2 infection: diagnosed based on positive nasal or throat swab virus antigen or nucleic acid test (19).

### 2.5 Statistical analysis

Statistical analysis was performed using IBM SPSS Statistics [version 26.0], Origin [version 9.9.0], GraphPad Prism [version 9.0.0], and RStudio [version 4.3.2]. Continuous variables between two groups were compared using t-tests for normally distributed variables, expressed as mean ± standard deviation. For non-normally distributed continuous variables, the Mann–Whitney U test was utilized and presented as the median (interquartile range). Categorical variables were represented by counts and percentages, with intergroup comparisons conducted using the chi-square test and Fisher’s exact test. The Spearman rank correlation analysis was employed to assess the correlation between opportunistic infections in IBD patients before and after SARS-CoV-2 infection. The software Origin and GraphPad Prism were utilized to visualize the combined opportunistic infection rates in IBD (UC and CD) patients before and after SARS-CoV-2 infection.

Utilizing both univariate and multivariate logistic regression analyses, we aimed to identify factors associated with opportunistic infections in IBD (UC and CD) patients before and after SARS-CoV-2 infection. Additionally, collinearity diagnosis was conducted for the multivariate logistic regression model, which resulted in rejecting its presence. Based on the multivariate logistic regression results (*p* < 0.05), nomogram models were separately constructed for predicting IBD (UC and CD) patients before and after SARS-CoV-2 infection using the R package rms in RStudio 4.3.2. Internal validation was performed using bootstrapping (1,000 iterations), and corresponding receiver operating characteristic (ROC) curves, calibration curves, clinical decision curves, and clinical impact curves were plotted to verify the accuracy and reliability of the prediction models. Multiple imputation was employed for continuous variables to fill missing values, limited to no more than 10%. All studies utilized two-tailed tests, with significant differences noted for *p* < 0.05.

## 3 Results

### 3.1 Demographic and clinical characteristics of IBD (UC and CD) patients before and after SARS-CoV-2 infection

This study included a total of 451 IBD patients (294 UC and 157 CD), among them, 207 cases (120 UC and 87 CD) were before SARS-CoV-2 infection, and 244 cases (174 UC and 70 CD) were after SARS-CoV-2 infection. The clinical and demographic features of IBD (UC and CD) both before and after SARS-CoV-2 infection are shown in Table 1. There were no statistically significant differences in baseline data between IBD (UC and CD) patients before and after SARS-CoV-2 infection, indicating a good balance and comparability of the two datasets.

**Table 1:** Demographic and clinical characteristics of IBD (UC and CD) patients before and after SARS-CoV-2 infection.

	IBD			UC			CD		
	Before SARS-CoV-2 infection	After SARS-CoV-2 infection	*p*-value	Before SARS-CoV-2 infection	After SARS-CoV-2 infection	*p*-value	Before SARS-CoV-2 infection	After SARS-CoV-2 infection	*p*-value
Patient (*n*)	207	244		120	174		87	70	
Age (years)	41.00 (31.00,54.00)	43.00 (33.00,56.00)	0.215	45.00 (34.00,57.75)	46.00 (36.00,57.00)	0.902	34.00 (25.00,47.00)	37.50 (27.75,51.50)	0.536
Height (cm)	170.00 (162.00,173.00)	168.00 (160.25,173.75)	0.404	168.50 (160.25,173.00)	168.00 (160.00,173.25)	0.939	170.00 (164.00,175.00)	168.00 (161.50,174.25)	0.248
Weight (kg)	50.00 (57.50,66.00)	58.00 (50.00,69.00)	0.441	57.75 (51.00,68.00)	60.00 (51.00,70.00)	0.379	57.00 (50.00,65.00)	55.00 (49.75,65.75)	0.756
BMI (kg/m^2^)	20.35 (18.54,22.91)	20.79 (19.03,23.44)	0.102	20.62 (18.64,23.63)	21.11 (19.36,23.53)	0.216	20.00 (18.29,22.22)	20.00 (18.22,22.56)	0.635
Duration of illness (years)	2.41 (0.34,5.17)	2.17 (0.41,5.98)	0.992	2.47 (0.34,5.25)	2.72 (0.80,6.35)	0.212	2.30 (0.36,4.92)	0.55 (0.00,2.50)	0.110
Gender *n* (%)			0.078			0.149			0.556
Male	138 (66.7)	143 (58.6)		77 (64.2)	97 (55.7)		61 (70.1)	46 (65.7)	
Female	69 (33.3)	101 (41.4)		43 (35.8)	77 (44.3)		26 (29.9)	24 (34.4)	
Disease activity *n* (%)			0.223			0.451			0.087
Active phase	189 (91.3)	230 (94.3)		116 (96.7)	165 (94.8)		14 (16.1)	5 (7.1)	
Remission phase	18 (8.7)	14 (5.7)		4 (3.3)	9 (5.2)		73 (83.9)	65 (92.9)	
Smoking *n* (%)	44 (21.3)	41 (16.8)	0.228	29 (24.2)	30 (17.2)	0.145	15 (17.2)	11 (15.7)	0.798
Alcohol consumption n (%)	46 (22.2)	38 (15.6)	0.071	32 (26.7)	31 (17.8)	0.069	14 (16.1)	7 (10.0)	0.265
Underlying chronic diseases *n* (%)	36 (17.4)	45 (18.4)	0.772	23 (19.2)	34 (19.5)	0.937	13 (14.9)	11 (15.7)	0.894
Extraintestinal manifestations *n* (%)	41 (19.8)	38 (15.6)	0.239	15 (12.5)	22 (12.6)	0.971	26 (29.9)	16 (22.9)	0.323
Previous medication use *n* (%)			<0.001			0.01			0.687
5-ASA	179 (44.6)	208 (58.3)		112 (49.3)	162 (63.0)		67 (38.7)	46 (46.0)	
Corticosteroids	94 (23.4)	69 (19.3)		62 (27.3)	54 (21.0)		32 (18.5)	15 (15.0)	
Immunosuppressants	46 (11.5)	19 (5.3)		22 (9.7)	7 (2.7)		23 (13.3)	12 (12.0)	
Biologics	82 (20.4)	61 (17.1)		31 (13.7)	34 (13.2)		51 (29.5)	27 (27.0)	

IBD, inflammatory bowel disease; UC, ulcerative colitis; CD, Crohn’s disease; BMI, body mass index.

### 3.2 The distribution and correlation of opportunistic infections in IBD (UC and CD) patients before and after SARS-CoV-2 infection

**Figure 2:**
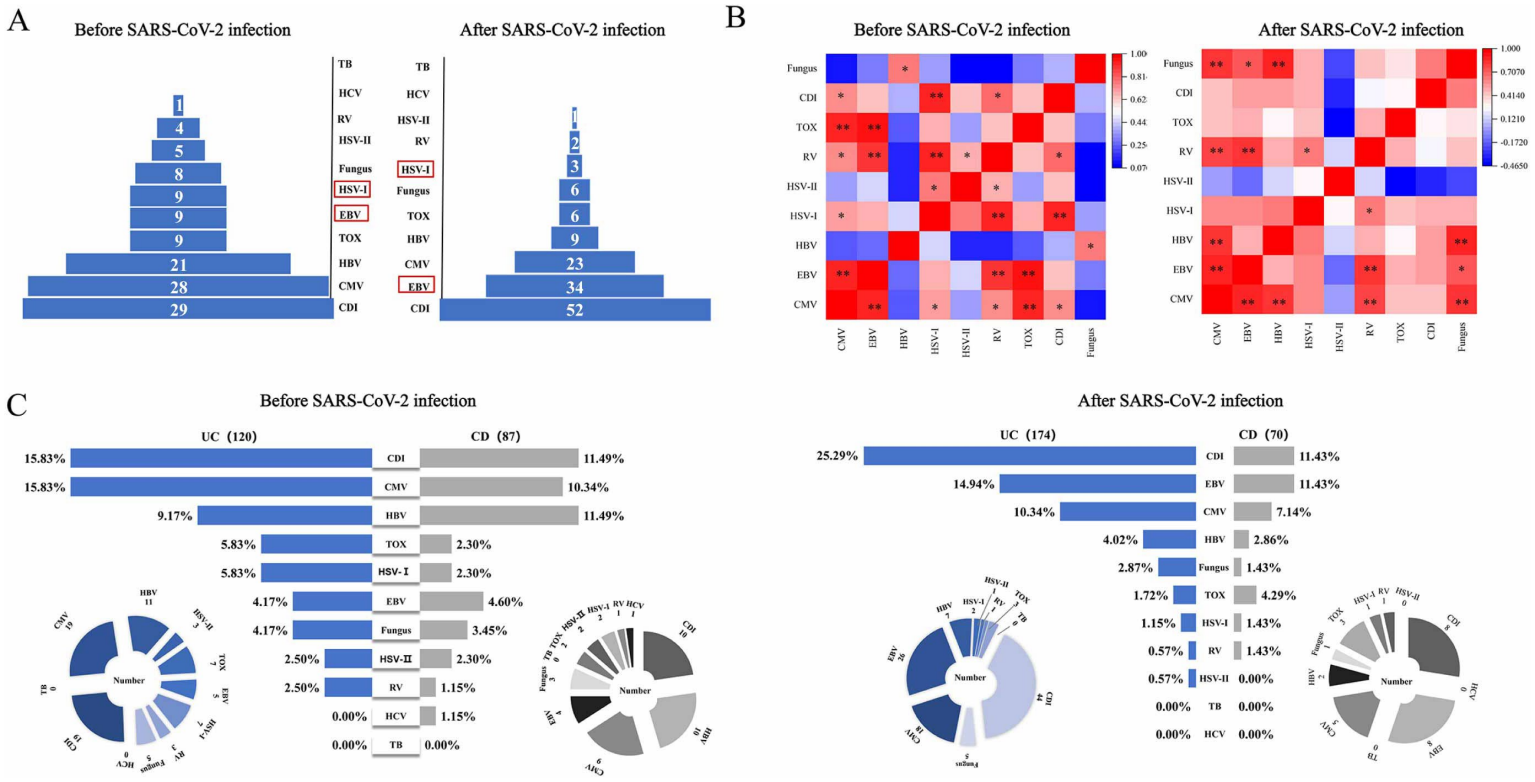
Changes in opportunistic pathogen infections in IBD patients before and after SARS-CoV-2 infection. **(A)** The spectrum of opportunistic infections in IBD patients; **(B)** heatmap of pathogen correlation, showing the correlation between different pathogens; **(C)** horizontal comparison of infection rates between UC and CD. [*p < 0.05, **p < 0.01]. UC, ulcerative colitis; CD, Crohn’s disease; CMV, cytomegalovirus, EBV, Epstein–Barr virus, HSV-I, herpes simplex virus I; HSV-II, herpes simplex virus II; HBV, hepatitis B virus; HCV, hepatitis C virus; RV, rubella virus; TB, tubercle bacilli; CDI, *Clostridium difficile* infection; TOX, toxoplasma.

The spectrum of opportunistic infections in IBD before and after SARS-CoV-2 infection. After infection, EBV levels rise, while HSV-I levels decrease, with CDI consistently ranking No. 1 infection (Figure 2A). Additionally, we constructed a correlation analysis matrix heatmap to assess the relationships between co-occurring opportunistic infections (Figure 2B). In the heatmap, red indicates a positive correlation, blue indicates a negative correlation, and the depth of color reflects the strength of the correlation. We found significant correlations (*p* < 0.01) persistently observed between CMV, RV, and EBV co-infections in contrast to before and after SARS-CoV-2 infection.

### 3.3 Comparison of the incidence of opportunistic infections in IBD (UC and CD) patients before and after SARS-CoV-2 infection

**Figure 3:**
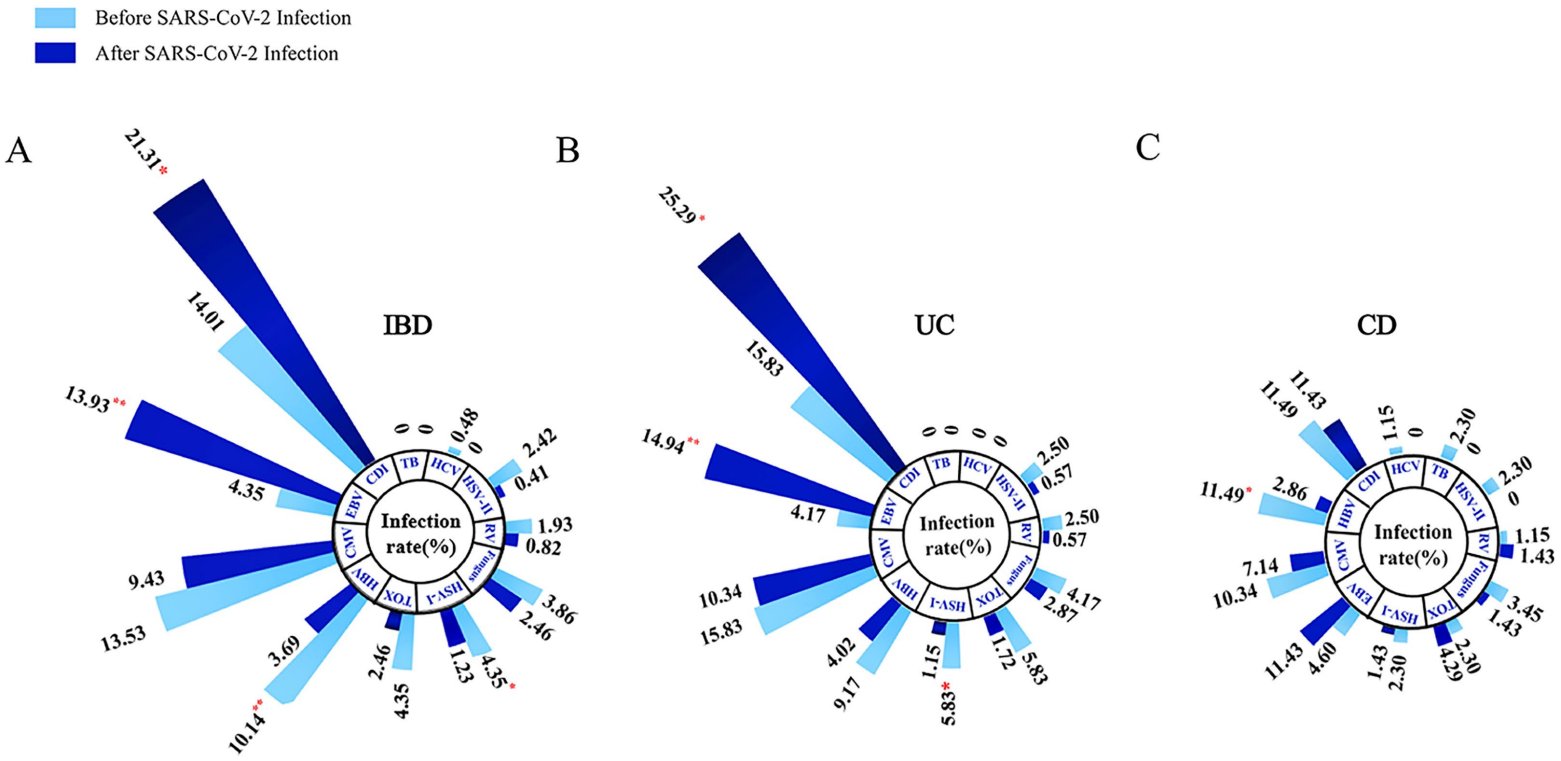
Longitudinal comparison of opportunistic infections complicating IBD **(A)**, UC **(B)**, and CD **(C)** patients before and after SARS-CoV-2 infection. [*p < 0.05, **p < 0.01]. IBD, inflammatory bowel disease; UC, ulcerative colitis; CD, Crohn’s disease; CMV, cytomegalovirus, EBV, Epstein-bar virus, HSV-I, herpes simplex virus I; HSV-II, herpes simplex virus II; HBV, hepatitis B virus; HCV, hepatitis C virus; RV, rubella virus; TB, tubercle bacilli; CDI, *Clostridium difficile* infection; TOX, toxoplasma.

We observed a significant increase in the incidence rates of CDI (21.31% vs. 14.01%, *p* = 0.044) and EBV (13.93% vs. 4.35%, *p* = 0.001) infections in IBD patients following SARS-CoV-2 infection, compared to pre-SARS-CoV-2 infection, indicating a statistically significant difference between the two. Conversely, the incidence rates of co-infections with HBV (3.69% vs. 10.14%, *p* = 0.006) and HSV-I (1.23% vs. 4.35%, *p* = 0.04) significantly decreased (Figure 3A).

Through longitudinal analysis of subgroups, we noted a significant increase in the incidence rates of CDI (25.29% vs. 15.83%, *p* = 0.049) and EBV (14.94% vs. 4.17%, *p* = 0.003) infections in UC patients following SARS-CoV-2 infection, while the incidence rate of co-infection with HSV-I significantly decreased (1.15% vs. 5.83%, *p* = 0.022; Figure 3B). For CD patients, we observed a significant decrease in the incidence rate of HBV co-infection following SARS-CoV-2 infection (2.86% vs. 11.49%, *p* = 0.043), with no significant differences in other aspects (Figure 3C). Subsequently, we further compared the lateral changes in infection rates between UC and CD patients before and after SARS-CoV-2 infection (Figure 2C) and found that UC patients had a higher incidence of opportunistic infections compared to CD patients. Additionally, regarding latent tuberculosis infection (LTBI) co-infection in IBD (UC and CD) patients before and after SARS-CoV-2 infection, we did not observe significant differences (Supplementary Figure 1).

### 3.4 Factors associated with the co-occurrence of opportunistic infections in IBD (UC and CD) patients before and after SARS-CoV-2 infection

In this study, variables with a *p*-value <0.05 from the univariate analysis of opportunistic infections in IBD patients before and after SARS-CoV-2 infection were included in the binary multivariable logistic regression model (Tables 2, 3). Before SARS-CoV-2 infection, duration of illness [Odds Ratio (OR): 1.082, 95% Confidence Interval (CI): 1.014–1.156, *p* = 0.018], red blood cells (RBC) [OR: 0.402, 95% CI: 0.268–0.603, *p* < 0.001], underlying chronic diseases [OR: 3.025, 95% CI: 1.195–7.656, *p* = 0.019], and alcohol consumption [OR: 6.916, 95% CI: 2.122–22.538, *p* = 0.001] were identified as factors associated with opportunistic infections in IBD patients (Table 4). On the other hand, after SARS-CoV-2 infection, corticosteroids use [OR: 3.082, 95% CI: 1.633–5.817, p = 0.001], RBC [OR: 0.529, 95% CI: 0.327–0.856, p = 0.01], total protein (TP) [OR: 0.948, 95% CI: 0.914–0.983, *p* = 0.004], and high-sensitivity C-reactive protein (Hs-CRP) [OR: 1.011, 95% CI: 1.002–1.020, *p* = 0.013] were identified as factors associated with opportunistic infections in IBD patients (Table 5).

**Table 2:** Univariate analysis of opportunistic infections complicating IBD patients before and after SARS-CoV-2 infection.

	Before SARS-CoV-2 infection			After SARS-CoV-2 infection		
	NCOI	COI	*p*-value	NCOI	COI	*p*-value
Patient (*n*)	133	74		151	93	
Age (years)	39.00 (30.00,55.00)	44.00 (34.75,54.00)	0.041	43.00 (33.00,54.00)	46.00 (33.00,58.00)	0.209
Height (cm)	170.00 (162.00,174.50)	169.00 (162.75,172.00)	0.389	167.72 ± 0.68	167.70 ± 0.85	0.983
Height (cm)	57.00 (50.00,68.00)	58.50 (51.75,65.25)	0.627	59.00 (50.00,70.00)	58.00 (50.50,65.00)	0.597
BMI (kg/m^2^)	20.20 (18.44,22.66)	20.79 (18.56,23.31)	0.431	21.16 (18.83,23.53)	20.57 (19.04,22.99)	0.330
Duration of illness (years)	2.10 (0.25,4.78)	2.80 (0.59,6.46)	0.031	2.19 (0.38,6.09)	1.84 (0.59,5.66)	0.875
Disease type *n* (%)			0.007	67 (44.4)	36 (38.7)	
UC	68 (51.1)	52 (70.3)		103 (68.2)	71 (76.3)	
CD	65 (48.9)	22 (29.7)		48 (31.8)	22 (23.7)	
Gender *n* (%)			0.473			0.229
Male	91 (68.4)	47 (63.5)		84 (55.6)	59 (63.4)	
Female	42 (31.6)	27 (36.5)		67 (44.4)	34 (36.6)	
Smoking *n* (%)	22 (16.5)	22 (29.7)	0.026	29 (19.2)	12 (12.9)	0.201
Alcohol consumption *n* (%)	18 (13.5)	28 (37.8)	<0.001	22 (14.6)	16 (17.2)	0.581
Underlying chronic diseases *n* (%)	18 (13.5)	18 (24.3)	0.050	27 (17.9)	18 (19.4)	0.773
Extraintestinal manifestations *n* (%)	25 (18.8)	16 (21.6)	0.625	25 (16.6)	13 (14.0)	0.590
Complications *n* (%)	29 (21.8)	10 (13.5)	0.144	16 (10.6)	12 (12.9)	0.583
Previous medication use *n* (%)						
5-ASA	111 (83.5)	68 (91.9)	0.089	130 (86.1)	78 (83.9)	0.635
Corticosteroids	60 (45.1)	34 (45.9)	0.908	29 (19.2)	40 (43.0)	<0.001
Immunosuppressants	23 (17.3)	23 (31.1)	0.022	8 (5.3)	11 (11.8)	0.065
Biologics	56 (42.1)	26 (35.1)	0.326	35 (23.2)	26 (28.0)	0.403
Disease activity *n* (%)			0.571			0.123
Remission phase	14 (10.5)	4 (5.4)		36 (23.8)	18 (19.4)	
Mild activity phase	25 (18.8)	17 (23.0)		71 (47.0)	40 (43.0)	
Moderate activity phase	44 (33.1)	23 (31.1)		33 (21.9)	32 (34.4)	
Severe activity phase	50 (37.6)	30 (40.5)		11 (7.3)	3 (3.2)	

COI, combined opportunistic infections; NCOI, not combined opportunistic infections; IBD, inflammatory bowel disease; UC, ulcerative colitis; CD, Crohn’s disease; BMI, body mass index. 5-ASA, 5-Aminosalicylic Acid.

**Table 3:** Univariate laboratory analysis of opportunistic infections complicating IBD patients before and after SARS-CoV-2 infection.

	Before SARS-CoV-2 infection			After SARS-CoV-2 infection		
	NCOI	COI	*p*-value	NCOI	COI	*p*-value
Patient (*n*)	133	74		151	93	
**Routine blood test**						
WBC (10^12^/L)	6.36 (4.98,8.31)	6.48 (4.93,8.43)	0.869	6.35 (4.94,7.75)	5.90 (4.70,8.10)	0.432
RBC (10^12^/L)	4.43 (3.93,4.93)	3.73 (2.81,4.40)	<0.001	4.47 (4.01,4.82)	4.28 (3.65,4.75)	<0.001
Hb (g/L)	124.00 (102.50,140.00)	122.00 (100.00,140.00)	0.615	125.00 (108.00,140.00)	117.00 (96.00,139.50)	0.351
Platelets (10^9^/L)	277.00 (191.00,351.50)	250.00 (205.75,345.00)	0.589	259.00 (198.00,334.00)	266.00 (207.50,345.00)	0.639
**Coagulation function**						
PT (S)	11.92 (10.95,13.05)	11.75 (10.80,12.93)	0.620	10.30 (9.02,11.80)	10.70 (9.48,11.90)	0.181
APTT (S)	31.00 (26.75,36.35)	31.10 (26.50,36.90)	0.792	31.00 (27.90,35.20)	31.50 (27.30,36.00)	0.994
TT (S)	16.33 (15.55,17.20)	16.30 (15.40,17.13)	0.685	17.00 (16.00,18.00)	16.80 (15.80,17.95)	0.384
Fibrinogen (g/L)	3.66 (2.66,4.50)	3.23 (2.48,4.81)	0.398	3.54 (2.72,4.71)	3.71 (2.96,4.83)	0.337
D-dimer (mg/L)	0.33 (0.22,0.75)	0.55 (0.28,1.06)	0.029	0.27 (0,15,0.64)	0.41 (0.16,0.98)	0.079
**Liver function test**						
ALT (IU/L)	15.00 (10.00,23.00)	16.50 (11.00,23.00)	0.268	14.00 (9.00,21.96)	15.00 (10.00,22.00)	0.658
AST (IU/L)	19.00 (15.00,25.00)	20.00 (15.75,25.25)	0.341	18.00 (13.00,23.00)	17.00 (14.0,23.00)	0.99
TBil (μ mol/L)	12.00 (8.65,17.85)	12.55 (8.28,17.25)	0.906	12.30 (8.80,17.10)	10.90 (8.45,14.15)	0.088
DBil (μ mol/L)	5.40 (3.00,7.80)	5.50 (2.70,7.73)	0.803	4.50 (3.10,7.00)	4.50 (3.10,7.80)	0.847
IBil (μ mol/L)	6.00 (4.40,10.70)	6.75 (4.65,9.93)	0.537	6.80 (4.76,9.20)	6.00 (4.05,7.65)	0.008
ALP (IU/L)	71.00 (60.00,84.00)	71.00 (57.00,87.25)	0.993	75.00 (61.00,92.00)	78.00 (60.50,96.00)	0.675
TP (g/L)	68.70 (63.75,72.75)	66.70 (60.10,74.05)	0.397	69.60 (64.60,73.50)	65.40 (57.70,71.65)	<0.001
**Renal function test**						
Urea (nmol/L)	4.20 (3.33,5.73)	4.34 (3.50,5.27)	0.850	4.24 (3.24,6.21)	4.36 (3.31,5.78)	0.926
Cr (μ mol/L)	64.00 (54.00,73.50)	61.00 (49.00,72.00)	0.137	60.00 (52.00,72.00)	61.45 (54.00,72.50)	0.292
**Trace elements**						
K (nmol/L)	3.95 (3.63,4.19)	3.87 (3.50,4.10)	0.122	3.92 (3.70,4.18)	3.83 (3.58,4.09)	0.098
Na (nmol/L)	140.80 (138.70,142.15)	140.30 (138.70,142.85)	0.581	141.20 (139.70,143.00)	141.60 (139.50,143.75)	0.522
Cl (nmol/L)	102.70 (100.25,105.45)	102.19 (99.55,104.90)	0.316	103.80 (102.30,105.60)	104.00 (101.60,105.85)	0.808
Ca (nmol/L)	2.14 (2.07,2.20)	2.11 (2.00,2.23)	0.169	2.17 (2.10,2.27)	2.14 (2.03,2.23)	0.021
CO_2_ (nmol/L)	25.23 ± 0.23	26.33 ± 0.37	0.158	24.69 (23.00,26.00)	25.10 (22.80,26.95)	0.302
Glu (mmol/L)	4.83 (4.43,5.29)	4.86 (4.55,5.37)		4.80 (4.52,6.23)	4.98 (4.36,6.23)	0.963
ESR (nm/h)	20.00 (9.50,37.50)	15.50 (8.00,44.75)	0.951	14.00 (7.00,35.00)	24.00 (12.00,39.00)	0.048
Hs-CRP (mg/L)	7.25 (1.66,20.50)	4.81 (1.21,19.99)	0.780	5.76 (0.93,33.20)	14.20 (2.70,38.00)	<0.001

IBD, inflammatory bowel disease; COI, combined opportunistic infections; NCOI, not combined opportunistic infections; WBC, white blood cell; RBC, red blood cell; Hb, hemoglobin; PT, prothrombin time; APTT, activated partial thromboplastin time; TT, thrombin time; ALT, alanine aminotransferase; AST, aspartate aminotransferase; TBil, total bilirubin; DBil, Direct Bilirubin; IBil, indirect bilirubin; ALP, Alkaline phosphatase; TP, total protein; Glu, glucose; ESR, erythrocyte sedimentation rate; Hs-CRP, hypersensitive C-reactive protein.

**Table 4:** Multifactorial analysis of opportunistic infections in patients with IBD before SARS-CoV-2 infection.

	*p*-value	OR (95%CI)
Age	0.324	0.986 (0.960, 1.014)
Duration of illness	0.018	1.082 (1.014, 1.156)
RBC	<0.001	0.402 (0.268, 0.603)
D-dimer	0.355	1.169 (0.839, 1.629)
Underlying chronic diseases *n* (%)	0.019	3.025 (1.195, 7.656)
Alcohol consumption *n* (%)	0.001	6.916 (2.122,22.538)
Smoking *n* (%)	0.286	0.516 (0.153, 1.739)
Immunosuppressants	0.408	1.401 (0.630, 3.116)

IBD, inflammatory bowel disease; RBC, red blood cell.

**Table 5:** Multifactorial analysis of opportunistic infections in patients with IBD after SARS-CoV-2 infection.

	*p*-value	OR (95%CI)
Corticosteroids	0.001	3.082 (1.633,5.817)
IBil	0.136	0.940 (0.867,1.020)
RBC	0.010	0.529 (0.327,0.856)
TP	0.004	0.948 (0.914,0.983)
Ca	0.433	0.848 (0.562,1.280)
ESR	0.230	0.990 (0.975,1.006)
Hs-CRP	0.013	1.011 (1.002,1.020)

IBD, inflammatory bowel disease; IBil, indirect bilirubin; RBC, red blood cell; TP, total protein; ESR, erythrocyte sedimentation rate; Hs-CRP, hypersensitive C-reactive protein.

Subgroup analyses were performed on IBD patients, with variables with a *p*-value<0.05 from the univariate analysis of UC (Supplementary Tables 1, 2) and CD (Supplementary Tables 5, 6) patients included in the multivariable logistic regression model. In UC patients, factors associated with the concurrent occurrence of opportunistic infections before SARS-CoV-2 infection included alcohol consumption [OR: 4.861, 95% CI: 1.769–13.357, *p* = 0.002], immunosuppressants [OR: 4.653, 95% CI: 1.319–16.416, *p* = 0.017], and RBC [OR: 0.486, 95% CI: 0.285–0.826, *p* = 0.008] (Supplementary Table 3). On the other hand, factors associated with the concurrent occurrence of opportunistic infections after SARS-CoV-2 infection in UC patients included disease extent E3 [OR: 6.361, 95% CI: 1.947–20.787, *p* = 0.002], RBC [OR: 0.352, 95% CI: 0.172–0.720, *p* = 0.004], TP [OR: 0.934, 95% CI: 0.889–0.981, *p* = 0.007], and high-sensitivity C-reactive protein (Hs-CRP) [OR: 1.017, 95% CI: 1.004–1.030, *p* = 0.011] (Supplementary Table 4). For CD patients, factors associated with the concurrent occurrence of opportunistic infections before SARS-CoV-2 infection included underlying chronic diseases [OR: 11.886, 95% CI: 1.841–76.736, *p* = 0.009], RBC [OR: 0.491, 95% CI: 0.261–0.924, *p* = 0.027], and D-dimer [OR: 5.125, 95% CI: 1.625–16.163, *p* = 0.005] (Supplementary Table 7). Factors associated with the concurrent occurrence of opportunistic infections after SARS-CoV-2 infection in CD patients included corticosteroids use [OR: 5.167, 95% CI: 1.261–21.170, *p* = 0.022] and immunosuppressants [OR: 7.333, 95% CI: 1.426–37.704, *p* = 0.017] (Supplementary Table 8). Collinearity diagnostics were conducted, and no multicollinearity was observed in the multivariable logistic regression models mentioned above. All variables had tolerance values greater than 0.10, and the variance inflation factors were less than 3.

### 3.5 Constructing a nomogram to predict the risk of opportunistic infections in IBD (UC and CD) before and after SARS-CoV-2 infection

**Figure 4:**
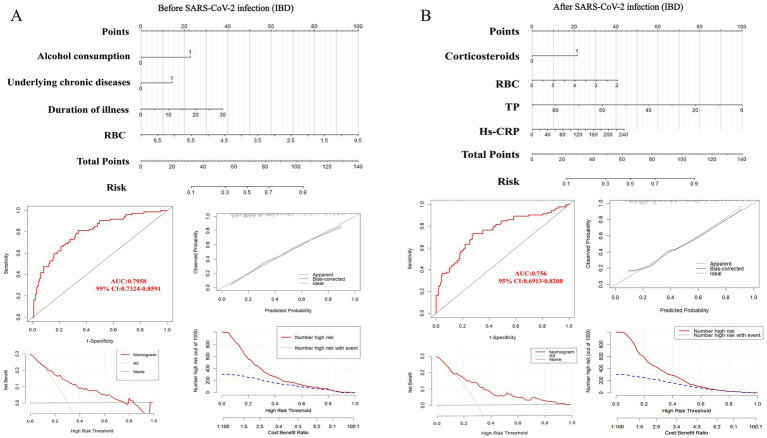
Nomogram predicting opportunistic infection risks complicating IBD before **(A)** and after **(B)** SARS-CoV-2 infection, along with ROC curve, clinical calibration curve, clinical decision curve, and clinical impact curve for model validation. IBD, inflammatory bowel disease; RBC, red blood cell. TP, total protein; Hs-CRP, hypersensitive C-reactive protein.

**Figure 5:**
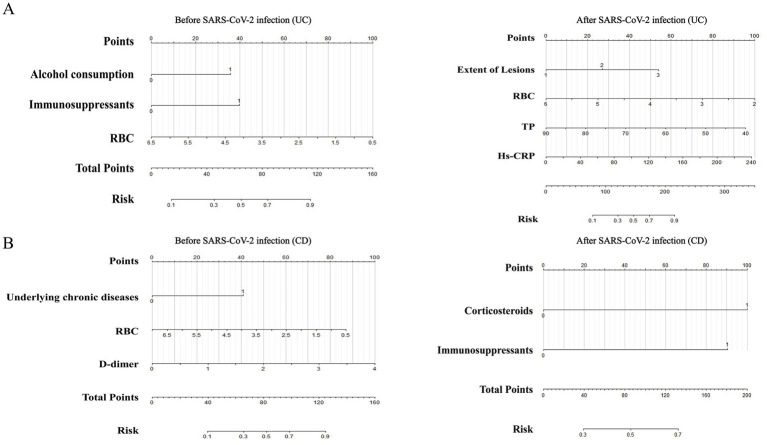
Nomograms predicting opportunistic infection risks complicating UC **(A)** and CD **(B)** before and after SARS-CoV-2 infection. UC, ulcerative colitis; CD, Crohn’s disease; RBC, red blood cell; TP, total protein; Hs-CRP, hypersensitive C-reactive protein.

We utilized the ‘rms’ package in R software to construct prediction models for IBD (Figure 4), UC, and CD (Figure 5) based on significant factors identified through multivariable logistic regression analysis (*p* < 0.05). In the nomogram for these models, points corresponding to each predictive variable on the scale are cumulatively added to derive a total score, which can be used to predict the risk of opportunistic infections.

To internally validate the reliability of the nomogram models, we first assessed their discriminative ability using ROC curves. The analysis showed that the AUC for IBD before SARS-CoV-2 infection (Figure 4A) was 0.7958 (95% CI: 0.7324–0.8591), with a cutoff value of 0.289 (sensitivity 0.811, specificity 0.662). In the UC model (Supplementary Figure 2A), the AUC was 0.7948 (95% CI: 0.7128–0.8768), with a cutoff value of 0.472 (sensitivity 0.635, specificity 0.853). The CD model (Supplementary Figure 3A) had an AUC of 0.8427 (95% CI: 0.7491–0.9362), with a cutoff value of 0.159 (sensitivity 0.909, specificity 0.723).

For models post-SARS-CoV-2 infection, the AUC for IBD (Figure 4B) was 0.8187 (95% CI: 0.7535–0.8838), with a cutoff value of 0.374 (sensitivity 0.731, specificity 0.722). The AUC for the UC model (Supplementary Figure 2B) was also 0.8187 (95% CI: 0.7535–0.8838), with a cutoff value of 0.413 (sensitivity 0.746, specificity 0.796). The CD model’s AUC (Supplementary Figure 3B) was 0.7254 (95% CI: 0.6025–0.8482), with a cutoff value of 0.384 (sensitivity 0.909, specificity 0.591). These results demonstrate the stability and high predictive accuracy of our models. Additionally, we evaluated the calibration of the models and examined the clinical decision and impact curves, which indicated good calibration and significant clinical utility.

## 4 Discussion

In this study, we examined the distribution and influencing factors of opportunistic infections in patients with IBD before and after SARS-CoV-2 infection. We further categorized the IBD patients into subgroups of UC and CD for subgroup analysis and developed predictive models for the risk of opportunistic infections before and after SARS-CoV-2 infection. Notably, this is the first study to explore the relationship between SARS-CoV-2 infection and the risk of opportunistic infections in IBD patients.

Our analysis revealed that SARS-CoV-2 infection significantly alters the risk of opportunistic infections in IBD patients. Specifically, after contracting SARS-CoV-2, the risk of infections like CDI and EBV increased, while the risk of HBV and HSV-I decreased, all with statistical significance. Additionally, although the risk of contracting other pathogens did not reach statistical significance, there was a noticeable decline compared to pre-infection levels, likely due to heightened health and hygiene vigilance during the pandemic. Further subgroup analysis revealed that UC patients generally had a higher risk of opportunistic infections than CD patients, consistent with findings from Zhou et al. (20). In UC patients, SARS-CoV-2 infection was positively correlated with increased risks of CDI and EBV infection but negatively correlated with HSV-I infection. This disparity could be attributed to increased antibiotic use following SARS-CoV-2 infection, which weakens and depletes the gut microbiome, thereby increasing susceptibility to CDI (21). Multiple studies have shown that SARS-CoV-2 infection facilitates the reactivation of EBV (22, 23), which aligns with our findings. We further observed that this phenomenon was particularly significant in UC patients. Moreover, according to Yan et al., SARS-CoV-2 infection may enhance the host’s immune memory cell response to HSV-I, reducing the risk of this infection (24), corroborating our observations. In CD patients, there was a negative correlation between SARS-CoV-2 infection and HBV infection risk. We speculate that this phenomenon might be linked to the declining prevalence of hepatitis viruses in China (25) and the reduction or cessation of hepatitis eradication programs and related interventions in hospitals during the pandemic (26, 27). Additionally, in IBD (UC and CD) patients, SARS-CoV-2 infection had no impact on LTBI. This might be due to disruptions in tuberculosis-related services due to national measures against SARS-CoV-2, thereby reducing the detection rates of active and latent tuberculosis infections. These findings are crucial for a deeper understanding of how SARS-CoV-2 infection affects the susceptibility to opportunistic infections in IBD patients and their prevention and control strategies.

After that, we conducted a detailed analysis of the factors associated with opportunistic infections in IBD patients before and after SARS-CoV-2 infection. On the one hand, before SARS-CoV-2 infection, the duration of the disease, red blood cell levels, underlying chronic diseases, and alcohol consumption were identified as predictors of opportunistic infections in IBD patients. Among these factors, disease duration is considered related to impaired immune function and prolonged exposure to healthcare environments (17). Additionally, reduced red blood cell levels, the presence of underlying chronic diseases, and alcohol consumption can all increase the burden on the immune system, weakening the body’s defenses against infections and making patients more susceptible to opportunistic infections (28–30). Subgroup analysis showed that for UC patients, alcohol consumption, red blood cell levels, and the use of immunosuppressants are closely related to the occurrence of opportunistic infections. For CD patients, however, underlying chronic diseases, red blood cell levels, and D-dimer levels are key factors. On the other hand, after SARS-CoV-2 infection, the use of corticosteroids, red blood cell levels, TP, and Hs-CRP became the main factors affecting opportunistic infections in IBD patients. Corticosteroid use can disrupt immune system function, thereby increasing the risk of opportunistic infections (31). The intestinal inflammation, absorption disorders, and release of inflammatory factors in IBD patients may lead to a decrease in TP and destruction of RBC, causing malnutrition and anemia (32, 33), which further impact immune function and increase the risk of opportunistic infections. Additionally, elevated levels of Hs-CRP indicate an aggravated inflammatory state, closely related to infection risk (34). Specifically, the factors related to opportunistic infections in UC patients include the extent of lesions, RBC, TP, and Hs-CRP, while for CD patients, they include corticosteroids and immunosuppressants.

Interestingly, our analysis shows that the correlation between the course of IBD, underlying chronic conditions, and alcohol consumption with opportunistic infections before SARS-CoV-2 infection does not persist post-infection. This observation suggests that the behaviors and medical management practices of this patient cohort may have changed during the pandemic. Particularly, IBD patients, especially those with a history of alcohol consumption, became more health-conscious post-SARS-CoV-2 infection due to their increased risk of severe COVID-19 (35), leading them to seek medical care more proactively, and many to choose abstinence from alcohol (36), which seems reasonable. Thus, enhanced medical attention and lifestyle changes may help reduce the incidence of opportunistic infections in this population post-SARS-CoV-2, a hypothesis consistent with other studies (37, 38). Notably, Soldera and colleagues also found that SARS-CoV-2 can not only trigger hemophagocytic lymphohistiocytosis in patients with IBD (39, 40), but also potentially increase the risk of complications related to primary sclerosing cholangitis (41–43). This highlights the importance of closely monitoring IBD patients for possible multisystem complications following SARS-CoV-2 infection, allowing for timely and effective intervention.

Currently, no model exists domestically or internationally that can predict the risk of opportunistic infections in IBD (UC and CD) patients before and after SARS-CoV-2 infection, which is concerning. Consequently, this study aims to construct a practical risk model with high predictive value to enable IBD patients to understand their potential risk of opportunistic infections early on. Through multivariable logistic regression analysis, we identified independent risk factors affecting IBD patients and incorporated these into a nomogram model to predict opportunistic infections before and after SARS-CoV-2 infection. This study further internally validated the model, assessing its discriminative ability using ROC curves, showing good stability and predictive accuracy. We also evaluated the model’s calibration and verified its clinical utility through decision curves and clinical impact curves.

However, this study has limitations. Due to the retrospective design of this study, there is a possibility of selection bias, which may result in insufficient representativeness of the sample and consequently affect the generalizability of the results. Additionally, the predictive model has not been externally validated, and future studies will need to use external datasets to confirm its reliability. Furthermore, since the data comes solely from China’s largest IBD research center, future studies should include large samples from different countries to validate the model’s applicability across different cultural contexts.

## 5 Conclusion

In conclusion, our study findings indicate that SARS-CoV-2 infection differently affects the risk and related factors for opportunistic infections in IBD (UC and CD) patients. The developed predictive model can provide decision support for clinicians and serve as a scientific basis for public health policy-making and the implementation of intervention measures.

## Data Availability

The original contributions presented in the study are included in the article/Supplementary material, further inquiries can be directed to the corresponding author.
