# Supplementary material for: Opportunistic infections changed before and after SARS-CoV-2 infection in inflammatory bowel disease patients: a retrospective single-center study in China
Source: Front Med (Lausanne). 2024 Sep 24;11:1461801. doi: 10.3389/fmed.2024.1461801 (PMC11460596; doi:10.3389/fmed.2024.1461801)
Supplement: Supplementary file 1 [file Data_Sheet_1.DOCX]

Supplementary Material

# Supplementary Figures and Tables

## Supplementary Figures
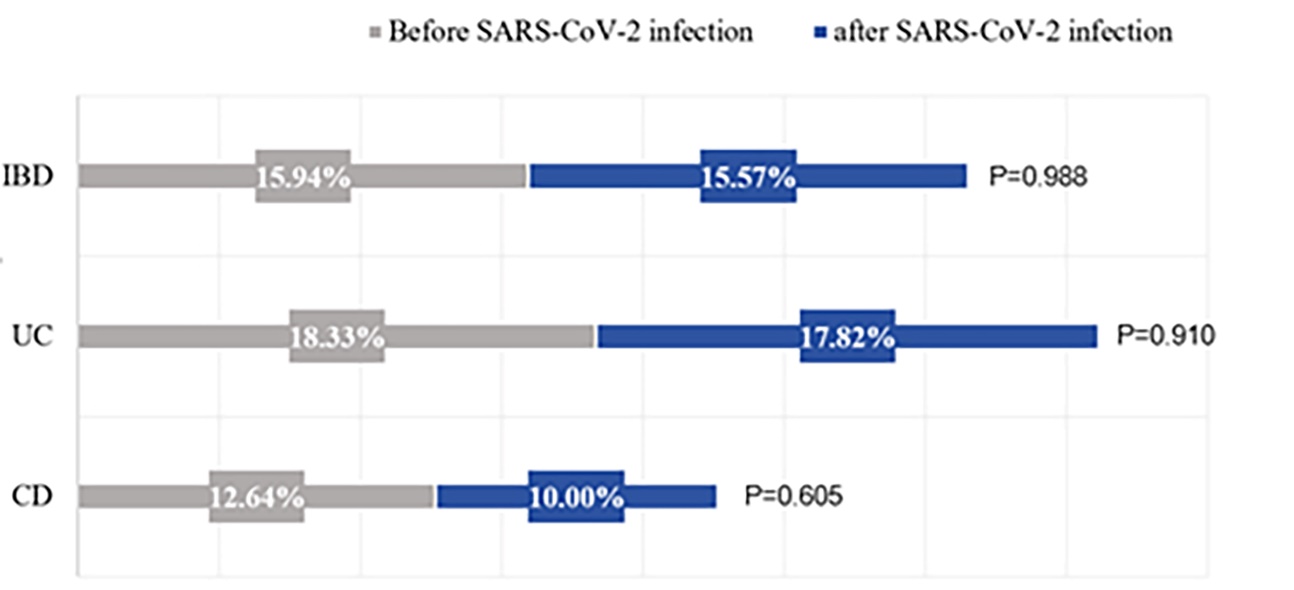


**Supplementary Figure 1.** Comparison of LTBI Complications in IBD (UC and CD) Before and After SARS-CoV-2 Infection. IBD, inflammatory bowel disease; UC, ulcerative colitis; CD, Crohn’s disease; LTBI: latent tuberculosis infection.


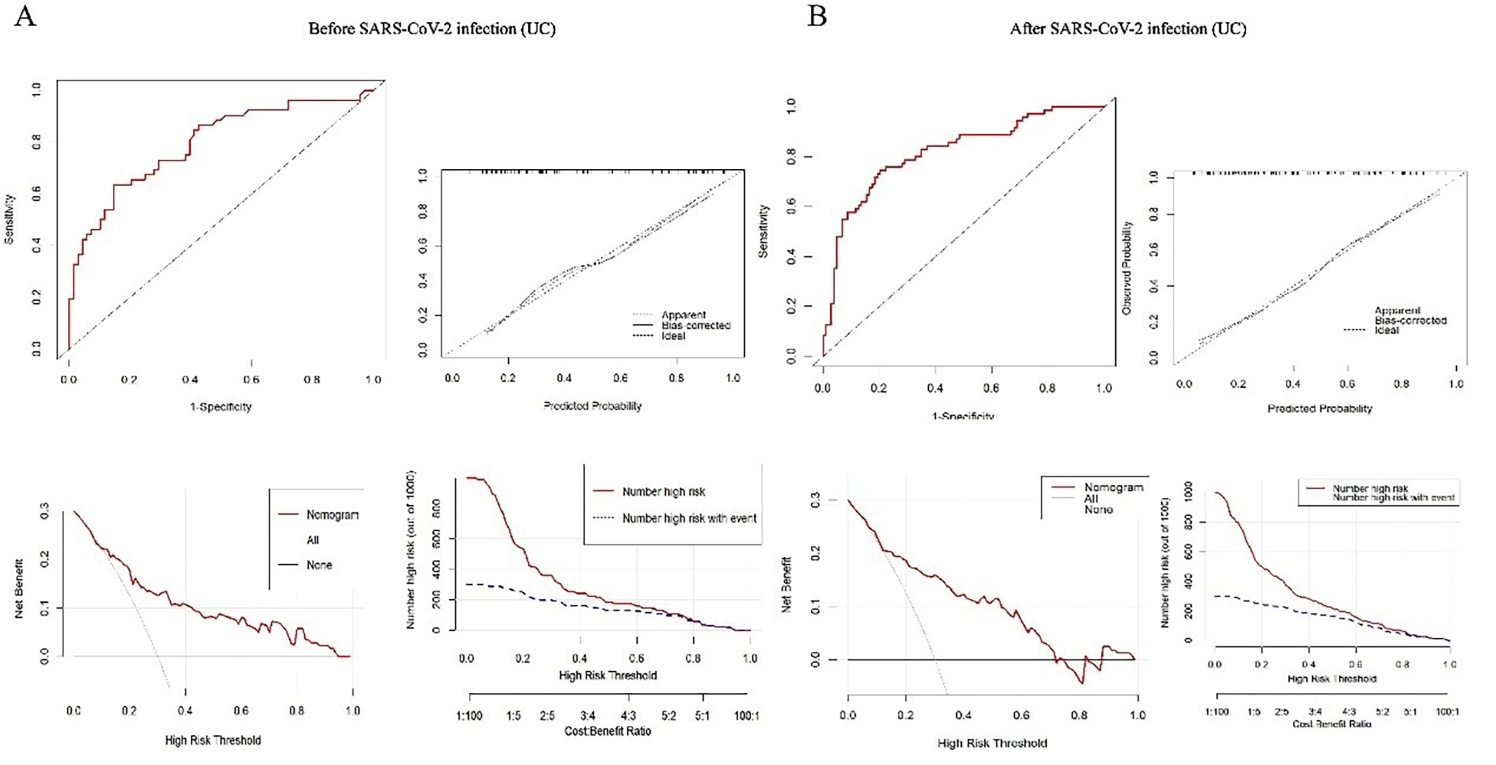


**Supplementary Figure 2.** ROC Curve, Clinical Calibration Curve, Clinical Decision Curve, and Clinical Impact Curve for Validating Opportunistic Infection Risk Models in UC Patients Before and After SARS-CoV-2 Infection (A, B). UC, ulcerative colitis.


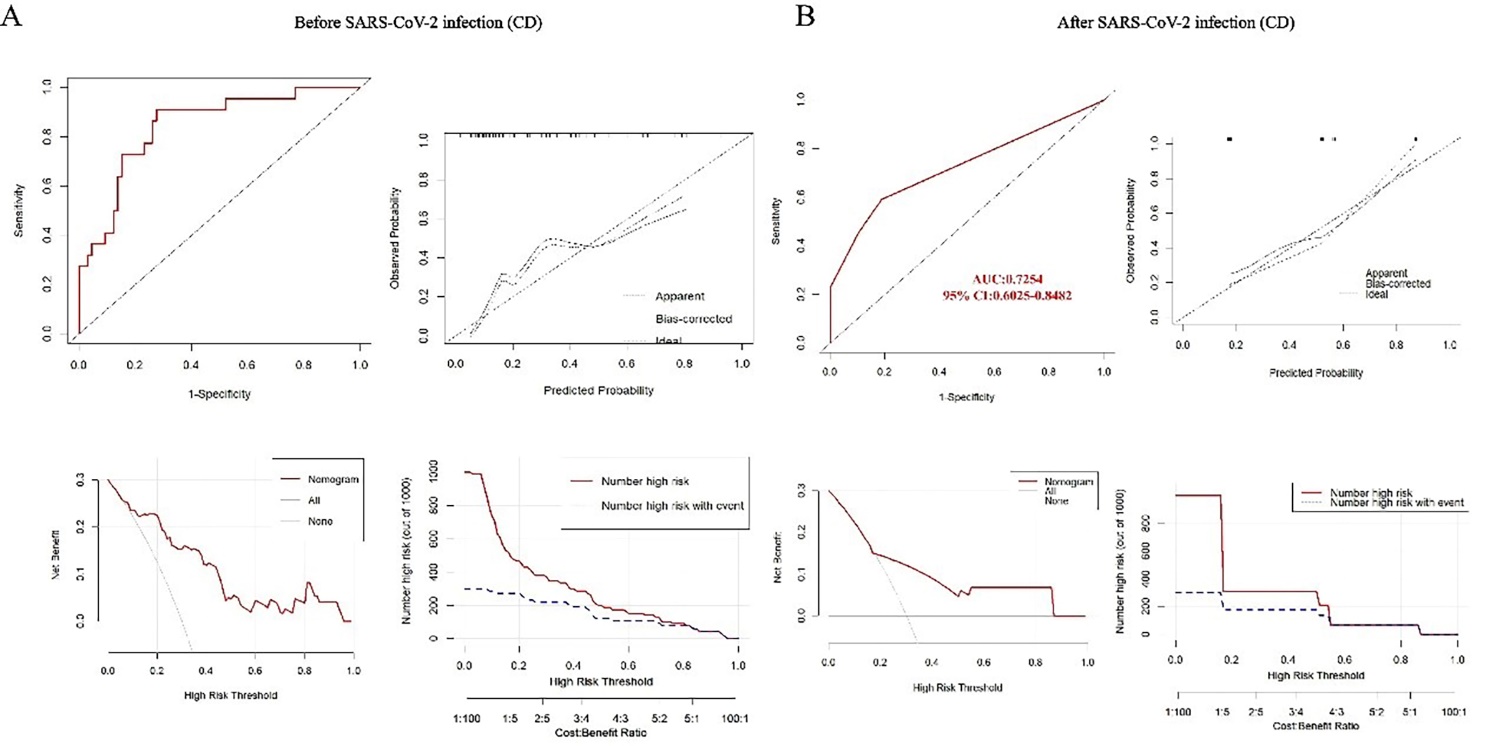


**Supplementary Figure 3.** ROC Curve, Clinical Calibration Curve, Clinical Decision Curve, and Clinical Impact Curve for Validating Opportunistic Infection Risk Models in CD Patients Before and After SARS-CoV-2 Infection (A, B). CD, Crohn’s disease.

## Supplementary Tables

**Supplementary Table 1**. Univariate Analysis of Opportunistic Infections Complicating UC Patients Before and After SARS-CoV-2 Infection.

|  | Before SARS-CoV-2 infection | | | After SARS-CoV-2 infection | | |
| --- | --- | --- | --- | --- | --- | --- |
|  | NCOI | COI | P | NCOI | COI | P |
| Patient (n) | 68 | 52 |  | 103 | 71 |  |
|  |  |  |  |  |  |  |
| Age (years) | 45.00(34.00,57.75) | 44.50(36.25,57.75) | 0.820 | 51.00(35.00,60.00) | 44.00(36.00,55.00) | 0.127 |
| Height (cm) | 167.87±1.01 | 167.54±0.90 | 0.814 | 168.00(161.00,174.00) | 168.00(160.00,173.00) | 0.712 |
| Weight (kg) | 55.50(50.00,68.75) | 60.00(53.00,68.00) | 0.209 | 60.00(51.00,70.00) | 59.00(51.00,65.00) | 0.299 |
| BMI（kg/m2） | 20.20(18.13,23.72) | 21.10(19.35,23.63) | 0.210 | 21.45(19.49,23.88) | 20.75(19.20,23.15) | 0.266 |
| Duration of illness (years) | 2.12(0.26,4.78) | 2.85(0.97,7.24) | 0.077 | 3.11(0.76,6.78) | 2.33(0.90,6.21) | 0.292 |
| Gender n (%) |  |  | 0.600 |  |  | 0.214 |
| Male | 45(66.2) | 32(61.5) |  | 53(51.5) | 44(62.0) |  |
| Female | 23(33.8) | 20(38.5) |  | 50(48.5) | 27(38.0) |  |
| Smoking n (%) | 13(19.1) | 16(30.8) | 0.140 | 19(18.4) | 11(15.5) | 0.177 |
| Alcohol consumption n (%) | 10(14.7) | 22(42.3) | 0.001 | 15(14.6) | 16(22.5) | 0.370 |
| Underlying chronic diseases n (%) | 13(19.1) | 10(19.2) | 0.988 | 21(20.4) | 13(18.3) | 0.734 |
| Extraintestinal manifestations n (%) | 10(14.7) | 5 (9.6) | 0.403 | 15(14.6) | 7 (9.9) | 0.359 |
| Complications n (%) | 6 (8.8) | 3 (5.8) | 0.730 | 6 (5.8) | 5 (7.0) | 0.760 |
| Previous medication use n (%) |  |  |  |  |  |  |
| 5-ASA | 62(91.2) | 50(96.2) | 0.463 | 98(95.1) | 64(90.1) | 0.233 |
| Corticosteroids | 37(54.4) | 25(48.1) | 0.491 | 24(23.3) | 30(42.3) | 0.008 |
| Immunosuppressants | 6 (8.8) | 16(30.8) | 0.004 | 4 (3.9) | 3 (4.2) | 0.910 |
| Biologics | 18(26.5) | 13(25.0) | 0.855 | 20(19.4) | 14(19.7) | 0.961 |
| Extent of Lesions n (%) |  |  | 0.703 |  |  | 0.027 |
| E1 | 7 (10.3) | 8 (15.4) |  | 31(30.1) | 11(15.5) |  |
| E2 | 24(35.3) | 17(32.7) |  | 37(35.9) | 23(32.4) |  |
| E3 | 37(54.4) | 27(51.9) |  | 35(34.0) | 37(52.1) |  |
| Disease activity n (%) |  |  | 0.940 |  |  | 0.038 |
| Remission phase | 2 (2.9) | 2 (3.8) |  | 26(25.2) | 12(16.9) |  |
| Mild activity phase | 8 (11.8) | 8 (15.4) |  | 44(42.7) | 29(40.8) |  |
| Moderate activity phase | 21(30.9) | 15(28.8) |  | 25(24.3) | 29(40.8) |  |
| Severe activity phase | 37(54.4) | 27(51.9) |  | 8 (7.8) | 1 (1.4) |  |

COI, combined opportunistic infections; NCOI, not combined opportunistic infections; UC, ulcerative colitis; BMI, body mass index; 5-ASA, 5-Aminosalicylic Acid; E1, proctitis; E2, Left-sided colitis; E3, Extensive colitis.

**Supplementary Table 2**: Univariate Analysis of Laboratory Test Results for Opportunistic Infections Complicating UC Patients Before and After SARS-CoV-2 Infection.

|  | Before SARS-CoV-2 infection | | | After SARS-CoV-2 infection | | |
| --- | --- | --- | --- | --- | --- | --- |
|  | NCOI | COI | P | NCOI | COI | P |
| Patient (n) | 68 | 52 |  | 103 | 71 |  |
| Routine Blood Test |  |  |  |  |  |  |
| WBC (10^12^/L) | 7.10(5.48,9.91) | 6.70(5.01,8.68) | 0.288 | 6.03(4.88,7.55) | 5.56(4.63,7.86) | 0.324 |
| RBC (10^12^/L) | 4.33(3.68,4.77) | 3.70(2.76,4.35) | 0.001 | 4.42(4.05,4.82) | 4.20(3.53,4.66) | <0.001 |
| Hb（g/L) | 118.88±3.12 | 119.92±3.90 | 0.833 | 127.00(111.00,141.00) | 116.00(92.00,138.00) | 0.194 |
| Platelets(10^9^/L) | 283.00(212.75,361.50) | 257.00(209.50,366.50) | 0.672 | 254.00(195.00,316.00) | 267.00(211.00,345.00) | 0.227 |
| Coagulation Function |  |  |  |  |  |  |
| PT（S） | 12.21±0.18 | 11.82±0.19 | 0.139 | 10.40(9.09,11.80) | 10.40(9.30,11.79) | 0.592 |
| APTT（S） | 31.80(26.72,36.88) | 29.60(26.10,36.13) | 0.430 | 30.70(27.90,34.50) | 30.69(27.10,35.70) | 0.758 |
| TT（S） | 16.40(15.73,17.38) | 16.20(15.23,17.08) | 0.195 | 17.10(16.00,18.00) | 17.00(15.70,18.00) | 0.533 |
| Fibrinogen(g/L) | 3.86(2.82,4.75) | 3.26(2.27,4.27) | 0.132 | 3.26(2.52,4.62) | 3.61(2.89,4.66) | 0.188 |
| D-dimer(mg/L) | 0.67(0.28,0.98) | 0.54(0.25,1.02) | 0.303 | 0.27(0.15,0.71) | 0.51(0.16,1.12) | 0.045 |
| Liver Function Test |  |  |  |  |  |  |
| ALT（IU/L） | 14.50(11.00,23.00) | 16.00(11.00,23.00) | 0.783 | 14.00(9.00,21.00) | 15.00(11.00,25.00) | 0.127 |
| AST（IU/L） | 19.00(15.00,25.00) | 19.50(15.00,24.00) | 0.968 | 18.00(13.00,23.00) | 19.00(14.00,24.00) | 0.306 |
| TBil（μmol/L） | 11.60(8.40,16.03) | 11.70(7.68,16.75) | 0.951 | 12.50(8.90,17.40) | 11.00(9.00,15.60) | 0.258 |
| DBil（μmol/L） | 5.40(2.95,7.68) | 4.80(2.43,6.98) | 0.244 | 4.50(3.10,7.40) | 5.00(3.20,7.80) | 0.585 |
| IBil（μmol/L） | 5.55(4.23,8.48) | 6.75(4.80,9.78) | 0.255 | 6.60(4.92,9.20) | 6.20(4.50,8.00) | 0.103 |
| ALP（IU/L） | 67.00(56.25,79.00) | 71.00(57.00,85.75) | 0.453 | 73.00(61.00,91.00) | 78.00(63.00,96.00) | 0.541 |
| TP（g/L） | 67.95(60.93,72.10) | 65.65(59.00,72.13) | 0.381 | 69.36±0.74 | 61.48±1.19 | <0.001 |
| Renal Function Test |  |  |  |  |  |  |
| Urea（nmol/L） | 4.37(3.35,5.81) | 4.26(3.46,5.03) | 0.494 | 4.25(3.37,6.20) | 4.67(3.47,6.04) | 0.490 |
| Cr（μmol/L） | 60.52±2.15 | 60.73±1.87 | 0.942 | 60.00(50.00,71.00) | 62.00(51.00,72.00) | 0.429 |
| Trace elements |  |  |  |  |  |  |
| K（nmol/L） | 3.97(3.61,4.22) | 3.87(3.49,4.03) | 0.129 | 3.89(3.61,4.15) | 3.77(3.53,4.18) | 0.200 |
| Na（nmol/L） | 140.70(138.10,141.88) | 140.25(138.70,143.08) | 0.966 | 141.30(139.70,143.10) | 141.20(139.60,143.70) | 0.842 |
| Cl（nmol/L） | 102.80(100.08,105.75) | 102.04(99.63,104.43) | 0.226 | 104.30(102.40,105.90) | 104.00(101.60,105.70) | 0.718 |
| Ca（nmol/L） | 2.13(2.03,2.20) | 2.11(1.97,2.18) | 0.371 | 2.18(2.10,2.28) | 2.10(2.00,2.24) | 0.020 |
| CO2（nmol/L） | 25.05(22.80,27.20) | 26.05(24.03,28.38) | 0.024 | 24.60(22.70,25.70) | 25.10(22.90,27.10) | 0.097 |
| Glu（mmol/L） | 4.88(4.43,5.44) | 4.79(4.56,5.34) | 0.863 | 4.80(4.52,6.23) | 4.99(4.49,6.23) | 0.803 |
| ESR（nm/h） | 24.50(12.00,36.25) | 15.00(8.00,32.75) | 0.144 | 13.00(7.00,28.00) | 24.00(12.00,30.00) | 0.025 |
| Hs-CRP（mg/L） | 6.62(1.32,19.79) | 4.81(1.32,16.10) | 0.851 | 3.68(0.80,20.00) | 13.10(2.61,47.70) | <0.001 |

UC, ulcerative colitis; COI, combined opportunistic infections; NCOI, not combined opportunistic infections; WBC, white blood cell; RBC, red blood cell; Hb, hemoglobin; PT, prothrombin time; APTT, activated partial thromboplastin time; TT, thrombin time; ALT, alanine aminotransferase; AST, aspartate aminotransferase; TBil, total bilirubin; DBil, Direct Bilirubin; IBil, indirect bilirubin; ALP, Alkaline phosphatase; TP, total protein; Glu, glucose; ESR, erythrocyte sedimentation rate; Hs-CRP, hypersensitive C-reactive protein.

**Supplementary Table 3.** Multivariate Analysis of Opportunistic Infections Complicating UC Patients Before SARS-CoV-2 Infection.

|  | P | OR (95%CI) |
| --- | --- | --- |
| Alcohol consumption | 0.002 | 4.861(1.769,13.357) |
| Immunosuppressants | 0.017 | 4.653(1.319,16.416) |
| RBC | 0.008 | 0.486(0.285, 0.826) |
| CO_2_ | 0.134 | 1.124(0.965, 1.308) |

UC, ulcerative colitis; RBC, red blood cell.

**Supplementary Table 4.** Multivariate Analysis of Opportunistic Infections Complicating UC Patients After SARS-CoV-2 Infection.

|  | P | OR (95%CI) |
| --- | --- | --- |
| Extent of Lesions |  |  |
| E1 |  | 1.00 |
| E2 | 0.141 | 2.415(0.747,7.805) |
| E3 | 0.002 | 6.361(1.947,20.787) |
| Disease activity |  |  |
| Remission phase |  |  |
| Mild activity phase | 0.176 | 5.480(0.466,64.480) |
| Moderate activity phase | 0.185 | 5.122(0.458,57.219) |
| Severe activity phase | 0.234 | 4.461(0.381,52.275) |
| Corticosteroids | 0.174 | 1.768(0.778,4.021) |
| RBC | 0.004 | 0.352(0.172,0.720) |
| TP | 0.007 | 0.934(0.889,0.981) |
| Ca | 0.268 | 0.817(0.572,1.168) |
| D-dimer | 0.756 | 0.987(0.906,1.075) |
| ESR（nm/h） | 0.101 | 0.979(0.954,1.004) |
| Hs-CRP（mg/L） | 0.011 | 1.017(1.004,1.030) |

UC, ulcerative colitis; E1, proctitis; E2, Left-sided colitis; E3, Extensive colitis; RBC, red blood cell.; TP, total protein; Glu, glucose; ESR, erythrocyte sedimentation rate; Hs-CRP, hypersensitive C-reactive protein.

**Supplementary Table 5.** Univariate Analysis of Opportunistic Infections Complicating CD Patients Before and After SARS-CoV-2 Infection.

|  | Before SARS-CoV-2 infection | | | After SARS-CoV-2 infection | | |
| --- | --- | --- | --- | --- | --- | --- |
|  | NCOI | COI | P | NCOI | COI | P |
| Patient (n) | 65 | 22 |  | 48 | 22 |  |
| Age (years) | 31.0(24.0,44.0) | 42.50(32.75,50.25) | 0.039 | 40.00(28.25,52.50) | 32.00(23.75,51.25) | 0.556 |
| Height (cm) | 169.15±1.04 | 168.23±1.68 | 0.650 | 167.04±1.31 | 168.82±1.69 | 0.433 |
| Weight (kg) | 58.90±1.38 | 55.40±2.26 | 0.203 | 55.00(49.00,69.50) | 55.50(50.00,65.00) | 0.653 |
| BMI（kg/m2） | 20.31(18.73,22.25) | 18.90(17.12,22.07) | 0.287 | 19.97(18.09,22.86) | 20.12(18.13,21.46) | 0.835 |
| Duration of illness (years) | 2.07(0.22,4.78) | 2.59(0.45,5.47) | 0.300 | 0.40(0.00,2.39) | 0.83(0.05,4.06) | 0.614 |
| Gender n (%) |  |  | 0.819 |  |  | 0.768 |
| Male | 46(70.8) | 15(68.2) |  | 31(64.6) | 15(68.2) |  |
| Female | 19(29.2) | 7 (31.8) |  | 17(35.4) | 7 (31.8) |  |
| Extent of Lesions n (%) |  |  | 0.219 |  |  | 0.838 |
| L1 | 33(50.8) | 9 (40.9) |  | 19(39.6) | 8 (36.4) |  |
| L2 | 10(15.4) | 8 (36.4) |  | 15(31.3) | 9 (40.9) |  |
| L3 | 21(32.3) | 5 (22.7) |  | 12(25.0) | 5 (22.7) |  |
| L4 | 1(1.5) | 0(0.0) |  | 2 (4.2) | 0 (0.0) |  |
| Disease behavior n (%) |  |  | 0.757 |  |  | 0.791 |
| B1 | 37(56.9) | 14(63.6) |  | 36(75.0) | 17(77.3) |  |
| B2 | 24(36.9) | 8 (36.4) |  | 11(22.9) | 5 (22.7) |  |
| B3 | 4 (6.2) | 0 (0.0) |  | 1 (2.1) | 0 (0.0) |  |
| Disease behavior n (%) |  |  |  |  |  |  |
| 5-ASA | 49(75.4) | 18(81.8) | 0.535 | 32(66.7) | 14(63.6) | 0.804 |
| Corticosteroids | 23(35.4) | 9 (40.9) | 0.642 | 5 (10.4) | 10(45.5) | 0.002 |
| Immunosuppressants | 19(29.2) | 4 (18.2) | 0.210 | 4 (8.3) | 8 (36.4) | 0.007 |
| Biologics | 38(58.5) | 13(59.1) | 0.959 | 15(31.3) | 12(54.5) | 0.063 |
| Disease activity n (%) |  |  | 0.522 |  |  | 0.786 |
| Remission phase | 12(18.5) | 2 (9.1) |  | 3 (6.3) | 2 (9.1) |  |
| Mild activity phase | 17(26.2) | 9 (40.9) |  | 10(20.8) | 6 (27.3) |  |
| Moderate activity phase | 23(35.4) | 8 (36.4) |  | 27(56.3) | 10(45.5) |  |
| Severe activity phase | 13(20.0) | 3 (13.6) |  | 8 (16.7) | 4 (18.2) |  |
| Smoking n (%) | 9 (13.8) | 6 (27.3) | 0.192 | 10(20.8) | 1 (4.5) | 0.154 |
| Alcohol consumption n (%) | 8 (12.3) | 6 (27.3) | 0.099 | 7 (14.6) | 0 (0.0) | 0.089 |
| Underlying chronic diseases n (%) | 5 (7.7) | 8 (36.4) | 0.003 | 6 (12.5) | 5 (22.7) | 0.303 |
| Extraintestinal manifestations n (%) | 15(23.1) | 11(50.0) | 0.017 | 10(20.8) | 6 (27.3) | 0.551 |
| Complications n (%) | 23(35.4) | 7 (31.8) | 0.761 | 10(20.8) | 7 (31.8) | 0.320 |

CD, Crohn’s disease; COI, combined opportunistic infections; NCOI, not combined opportunistic infections; BMI, body mass index; 5- ASA, 5-Aminosalicylic Acid; L1, terminal ileum; L2, colon; L3, ileum colon; L4, upper gastrointestinal tract; B1, non-narrow, non-fistula; B2, narrow; B3, fistula.

|  | Before SARS-CoV-2 infection | | | After SARS-CoV-2 infection | | |
| --- | --- | --- | --- | --- | --- | --- |
|  | NCOI | COI | P | NCOI | COI | P |
| Patient (n) | 65 | 22 |  | 48 | 22 |  |
| Routine Blood Test |  |  |  |  |  |  |
| WBC (1012/L) | 5.99(4.44,7.31) | 6.39(4.55,7.16) | 0.718 | 6.64(5.29,8.03) | 6.76(5.06,9.01) | 0.578 |
| RBC (1012/L) | 4.58(4.01,5.11) | 3.92(2.80,4.46) | 0.003 | 4.39±0.10 | 4.34±0.17 | 0.820 |
| Hb（g/L) | 124.00(102.00,139.00) | 112.00(100.00,137.50) | 0.161 | 119.55(105.25,133.00) | 122.50(108.50,141.50) | 0.502 |
| Platelets(109/L) | 255.00(184.00,327.00) | 231.00(192.50,299.75) | 0.348 | 278.17(198.25,388.50) | 260.00(174.50,335.25) | 0.318 |
| Coagulation Function |  |  |  |  |  |  |
| PT（S） | 11.70(10.90,12.90) | 12.50(10.80,13.5) | 0.458 | 10.25(8.99,11.80) | 11.35(9.74,12.63) | 0.089 |
| APTT（S） | 31.82±0.80 | 34.35±1.78 | 0.064 | 31.80(27.77,36.20) | 33.15(27.48,37.98) | 0.544 |
| TT（S） | 16.30(15.50,17.20) | 16.45(15.80,17.43) | 0.213 | 17.00(16.03,17.98) | 16.80(16.05,17.53) | 0.523 |
| Fibrinogen(g/L) | 3.35(2.61,4.35) | 3.11(2.81,5.09) | 0.605 | 3.40(3.29,5.45) | 4.27(3.19,5.33) | 0.789 |
| D-dimer(mg/L) | 0.26(0.17,0.36) | 0.62(0.31,1.47) | 0.0001 | 0.27(0.15,0.61) | 0.23(0.16,0.41) | 0.699 |
| Liver Function Test |  |  |  |  |  |  |
| ALT（IU/L） | 15.00(11.00,22.00) | 17.50(12.00,23.25) | 0.213 | 13.00(10.00,23.50) | 10.50(5.75,18.50） | 0.057 |
| AST（IU/L） | 20.00(15.00,25.00) | 20.50(17.75,28.00) | 0.106 | 17.00(13.25,22.80) | 14.50(10.00,17.00) | 0.039 |
| TBil（μmol/L） | 13.40(9.00,19.10) | 15.35(9.93,21.93) | 0.312 | 12.02(8.33,16.10) | 9.80(7.55,13.80) | 0.146 |
| DBil（μmol/L） | 6.30(3.40,8.80) | 7.00(4.40,8.93) | 0.110 | 4.90(3.13,6.88) | 3.60(2.55,5.95) | 0.362 |
| IBil（μmol/L） | 6.80(4.80,11.70) | 6.75(3.95,13.38) | 0.922 | 7.10(4.56,8.75) | 5.65(3.40,6.50) | 0.045 |
| ALP（IU/L） | 77.00(63.00,91.00) | 73.50(57.00,93.50) | 0.784 | 76.62(60.25,94.00) | 80.50(56.75,97.75) | 0.929 |
| TP（g/L） | 70.00(65.80,73.60) | 70.25(66.70,76.00) | 0.467 | 68.60(64.98,72.73) | 68.55(62.40,71.88) | 0.617 |
| Renal Function Test |  |  |  |  |  |  |
| Urea（nmol/L） | 4.25(3.31,5.73) | 4.57(3.65,6.54) | 0.133 | 3.89(3.13,6.53) | 3.81(3.11,4.84) | 0.302 |
| Cr（μmol/L） | 66.00(53.00,74.00) | 53.50(46.00,71.00) | 0.080 | 61.57±1.96 | 64.49±3.41 | 0.433 |
| Trace elements |  |  |  |  |  |  |
| K（nmol/L） | 3.90(3.65,4.17) | 3.57(3.91,4,21) | 0.781 | 3.97(3.80,4.23) | 3.95(3.79,4.06) | 0.463 |
| Na（nmol/L） | 141.10(138.80,142.60) | 138.80(140.85,141.80) | 0.432 | 141.06±0.40 | 141.92±0.67 | 0.256 |
| Cl（nmol/L） | 102.39±0.43） | 102.29±0.93 | 0.889 | 103.55(101.78,105.33) | 103.75(101.40,107.73) | 0.479 |
| Ca（nmol/L） | 2.14(2.06.2.23) | 2.05(2.10,2.24) | 0.522 | 2.17±0.02 | 2.15±0.02 | 0.495 |
| CO2（nmol/L） | 25.62±0.29 | 26.14±0.71 | 0.307 | 24.90±0.32 | 24.14±0.64 | 0.241 |
| Glu（mmol/L） | 4.78(4.44,5.27) | 4.96(4.44,5.51) | 0.529 | 4.84(4.54,6.23) | 4.90(4.06,6.23) | 0.528 |
| ESR（nm/h） | 15.00(8.00,40.00) | 30.50(11.00,86.00) | 0.105 | 24.50(12.00,44.50) | 25.00(15.00,65.00) | 0.436 |
| Hs-CRP（mg/L） | 7.50(1.90,24.55) | 5.37(1.21,22.20) | 0.903 | 14.35(3.39,48.04) | 19.65(7.22,39.30) | 0.448 |

**Supplementary Table 6.** Univariate Analysis of Laboratory Test Results for Opportunistic Infections Complicating CD Patients Before and After SARS-CoV-2 Infection.

CD, Crohn’s disease; COI, combined opportunistic infections; NCOI, not combined opportunistic infections; WBC, white blood cell; RBC, red blood cell; Hb, hemoglobin; PT, prothrombin time; APTT, activated partial thromboplastin time; TT, thrombin time; ALT, alanine aminotransferase; AST, aspartate aminotransferase; TBil, total bilirubin; DBil, Direct Bilirubin; IBil, indirect bilirubin; ALP, Alkaline phosphatase; TP, total protein; Glu, glucose; ESR, erythrocyte sedimentation rate; Hs-CRP, hypersensitive C-reactive protein.

**Supplementary Table 7.** Multivariate Analysis of Opportunistic Infections Complicating CD Patients Before SARS-CoV-2 Infection.

|  | P | OR (95%CI) |
| --- | --- | --- |
| Age | 0.226 | 0.966 (0.915, 1.021) |
| Underlying chronic diseases n (%) | 0.009 | 11.886(1.841,76.736) |
| Extraintestinal manifestations | 0.217 | 2.312 (0.611, 8.754) |
| RBC | 0.027 | 0.491 (0.261, 0.924) |
| D-dimer | 0.005 | 5.125 (1.625,16.163) |

CD, Crohn’s disease; RBC, red blood cell.

**Supplementary Table 8.** Multivariate Analysis of Opportunistic Infections Complicating CD Patients After SARS-CoV-2 Infection.

|  | P | OR (95%CI) |
| --- | --- | --- |
| Corticosteroids | 0.022 | 5.167(1.261,21.170) |
| Immunosuppressants | 0.017 | 7.333(1.426,37.704) |
| AST | 0.246 | 0.935(0.834, 1.048) |
| IBil | 0.369 | 0.928(0.788, 1.093) |

CD, Crohn’s disease; AST, aspartate aminotransferase; IBil, indirect bilirubin.
